# [^18^F]VM4-037 MicroPET Imaging and Biodistribution of Two *In Vivo* CAIX-Expressing Tumor Models

**DOI:** 10.1007/s11307-015-0831-y

**Published:** 2015-02-24

**Authors:** Sarah G. J. A. Peeters, Ludwig Dubois, Natasja G. Lieuwes, Dennis Laan, Martien Mooijer, Robert C. Schuit, Daniela Vullo, Claudiu T. Supuran, Jonas Eriksson, Albert D. Windhorst, Philippe Lambin

**Affiliations:** 1 Department of Radiation Oncology (MaastRO Lab), GROW—School for Oncology and Developmental Biology, Maastricht University Medical Centre, UNS 50/23, PO Box 616, 6200 MD Maastricht, The Netherlands; 2 Department of Radiology and Nuclear Medicine, VU University Medical Center, De Boelelaan 1117, PO Box 7057, 1081 HV Amsterdam, The Netherlands; 3 Neurofarba Department, Sezione di Scienze Farmaceutiche and Laboratorio di Chimica Bioinorganica, Università degli Studi di Firenze, Rm. 188, Via della Lastruccia 3, 50019 Sesto Fiorentino, Florence Italy

**Keywords:** [^18^F]VM4-037, CAIX, PET imaging, Biodistribution, Preclinical

## Abstract

**Purpose:**

[^18^F]VM4-037 was recently developed as a positron emission tomography (PET) tracer for the detection of carbonic anhydrase IX (CAIX), a tumor-specific protein upregulated under hypoxic conditions. In this study, the accumulation of [^18^F]VM4-037 was determined in two CAIX-expressing preclinical human tumor models.

**Procedures:**

U373 and HT29 tumor-bearing animals were injected with [^18^F]VM4-037 and underwent microPET imaging up to 4 h post-injection (p.i.). Biodistribution throughout the different organs was assessed at 2 and 4 h p.i. using gamma counting.

**Results:**

MicroPET imaging showed high [^18^F]VM4-037 uptake in the abdominal region, and biodistribution revealed high radioactivity in the kidney, ileum, colon, liver, stomach, and bladder. Although high CAIX expression was confirmed in both tumor models, tumor uptake assessed with microPET and biodistribution experiments was comparable to background tissues.

**Conclusions:**

In this study, [^18^F]VM4-037 does not specifically accumulate in CAIX-expressing tumors, indicating that the tracer is not suitable for the detection of CAIX.

## Introduction

Carbonic anhydrase IX (CAIX) is a tumor-specific protein upregulated in response to hypoxia [1]. Low oxygen concentrations are a common phenomenon in solid tumors [2]. One of the key adaptations to this condition is the stabilization of “hypoxia-inducible factor” (HIF) [3], which regulates the transcription of several genes including the *CA9* gene [1]. CAIX plays a main role in the regulation of the cellular pH. By catalyzing the reaction of CO_2_ and H_2_O to bicarbonate and a proton, CAIX preserves the stable intracellular environment and acidifies the extracellular environment [4]. Furthermore, the expression of CAIX has been found to be a negative predictor for disease-free survival [5]. Inhibition of CAIX function by targeted drugs would therefore be a promising anticancer approach.

Several specific CAIX inhibitors have been developed and described [6]. Recent publications show that inhibition of CAIX results in reduced tumor growth *in vivo*, especially in combination with radiotherapy [7, 8]. Furthermore, *in vitro* and *in vivo* results indicate the potential involvement of CAIX in processes like metastasis and invasion [9–11]. Before clinical implementation of these specific inhibitors, it would be extremely valuable to have a screening method to assess the CAIX status of tumors. This would allow patient selection before the start of treatment. A new promising imaging radiopharmaceutical, [^18^F]VM4-037, has recently been proposed. So far, this radiopharmaceutical has been tested in healthy human volunteers, demonstrating the safety of [^18^F]VM4-037 [12]. We assessed the tumor-specific uptake of this positron emission tomography (PET) radiopharmaceutical in two preclinical CAIX-expressing tumor models using microPET and biodistribution.

## Material and Methods

### Synthesis of [^18^F]VM4-037

Synthesis of radioactive-labeled (*S*)-3-(4-(2-[^18^F]fluoroethoxy)phenyl)-2-(3-methyl-2-(4-((2-sulfamoylbenzo[*d*]thiazol-6-yloxy)methyl)-1*H*-1,2,3-triazol-1-yl)butanamido) propanoic acid, also referred to as [^18^F]VM4-037, was performed as described previously [12]. The high-performance liquid chromatography (HPLC)-isolated [^18^F]VM4-037 fractions demonstrated a radiochemical purity always >99 %. Specific activity was typically in the range of 15–50 GBq/μmol and the decay-corrected synthesis yield was 7–9 %.

### Animal and Tumor Model, PET Acquisition, and Metabolism

All animal experiments were approved by the ethical committee of the local universities. All applicable institutional and national guidelines for the care and use of animals were followed. U373 glioma (ATCC HTB-17) or HT29 (ATCC HTB-38) colorectal adenocarcinoma cells, resuspended in Basement Membrane Matrix (Matrigel^TM^ BD Biosciences), were subcutaneously injected into the lateral flank or the shoulder of NMRI (nu/nu) mice. Tumor volume was measured on a regular basis using a Vernier caliper and calculated by the formula *A* × *B* × *C* × *π*/6 in which *A*, *B*, and *C* are the three orthogonal diameters of the tumor, each corrected for the thickness of the skin. The minimum tumor volume for inclusion was >300 mm^3^ to ensure clear visualization on the microPET. Upon an average tumor volume of 597 mm^3^ (range 358–981) and 1192 mm^3^ (range 463–1745) for U373 (*n* = 4) and HT29 (*n* = 3), respectively, PET acquisition was performed under isoflurane anesthesia (induction 4 %, maintenance 1–2 %).

PET imaging was performed using the Focus 120 microPET (Siemens Medical Solutions USA, Inc.) with an axial field of view (FOV) of 7.6 cm, a transaxial FOV of 10 cm, and a resolution of approximately 1.5 mm. The animals were positioned in the center of the FOV of the microPET using a laser-positioning system.

A bolus injection of [^18^F]VM4-037 (3.79 ± 0.55 MBq) was given via a catheter placed in the lateral tail vein. PET acquisitions for the U373 tumor model (*n* = 4 mice, all bearing a tumor) were performed at time points 0, 120, and 240 min post-injection (p.i.) using a 15-min emission scan. Image acquirement of the HT29 tumor model (*n* = 4 mice, three of which had a tumor) was performed at 30, 60, 90, and 120 min after injection using a 10-min emission scan. Each scan was corrected for random counts, dead time, and decay. List mode acquisition and reconstruction were performed as previously described [13]. Images were visualized and analyzed using ASIPro VM software (version 6.3.3.0).

### Biodistribution

After the final PET acquisition, animals were sacrificed and prepared for biodistribution. The tumor, blood, skin, bladder, heart, lungs, liver, spleen, stomach, ileum, colon, kidney, lymph nodes, salivary gland, muscle, and brain were weighed, and the amount of radioactivity in each organ was assessed using a gamma-well counter (1480 Wallac Wizard 3″ automatic γ-counter; PerkinElmer, Inc.). Tumors were halved, after which one piece was used for gamma counting and the other half was snap frozen for protein isolation. Disintegrations were counted for 1 min for each sample using an energy window of 350 and 650 keV. Samples were corrected for background and decay, and the percentage of the injected dose per gram (%ID/g) was calculated.

### Metabolite Analysis

C57BL/6 mice were injected with [^18^F]VM4-037 (21.27 ± 0.42 MBq). At 15 min (*n* = 3) and 45 min (*n* = 3), animals were sacrificed and blood was collected via a heart puncture, centrifuged (4 min, 4000 rpm), and plasma was harvested and diluted with acidified water. An activated (10 ml of ethanol followed by 10 ml of water) Waters tC18 Sep-Pak solid phase extraction cartridge was used to separate the polar and apolar fractions, which were further analyzed with HPLC (Dionex UltiMate 3000), using a Phenomenex Gemini 5 μ, 250 × 10 column as stationary phase. The mobile phase consisted of acetonitrile (A) and a 90 to 15 % gradient of 10 mM phosphoric acid (B) at a flow of 3 ml/min.

### Western Blotting

Tumors were minced and protein was isolated using RIPA buffer completed with a protease inhibitor cocktail (complete EDTA-free; Roche, Indianapolis, USA). Protein concentrations were determined by Bradford assay (BioRad, Veenendaal, Netherlands) and protein was separated on a 10 % SDS-PAGE gel. After blotting onto a nitrocellulose membrane (GE Healthcare, Amersham Corp, UK) by electrotransfer, membranes were blocked in 5 % non-fat dry milk and probed overnight with antibodies against CAIX (M75, kindly provided by S. Pastorekova, Institute of Virology, Slovak Academy of Science, Bratislava, Slovak Republic) and β-actin (Cell Signaling). Secondary antibodies (α-mouse IgG, Cell Signaling) were detected with Western blot detection reagents (Thermo Scientific Pierce, Rockford, USA).

### Delta pH Assay

HT29 cells were seeded and incubated for 24 h under hypoxic conditions (0.2 % O_2_, 5 % CO_2_, residual N_2_; MACS VA500 microaerophilic workstation, Don Whitley Scientific, Shipley, UK) with the addition of either vehicle (1 % DMSO), VM4-037 (1 mM), or 4-(2-aminoethyl)benzene sulfonamide (AEBS; 1 mM), a positive control for CAIX inhibition (*n* = 4) [4]. The pH of the culture medium was immediately measured at the end of each experiment, as described in a previous study [14]. The inhibitory constant (Ki) were obtained with a stopped-flow assay method [15].

## Results

**Fig. 1:**
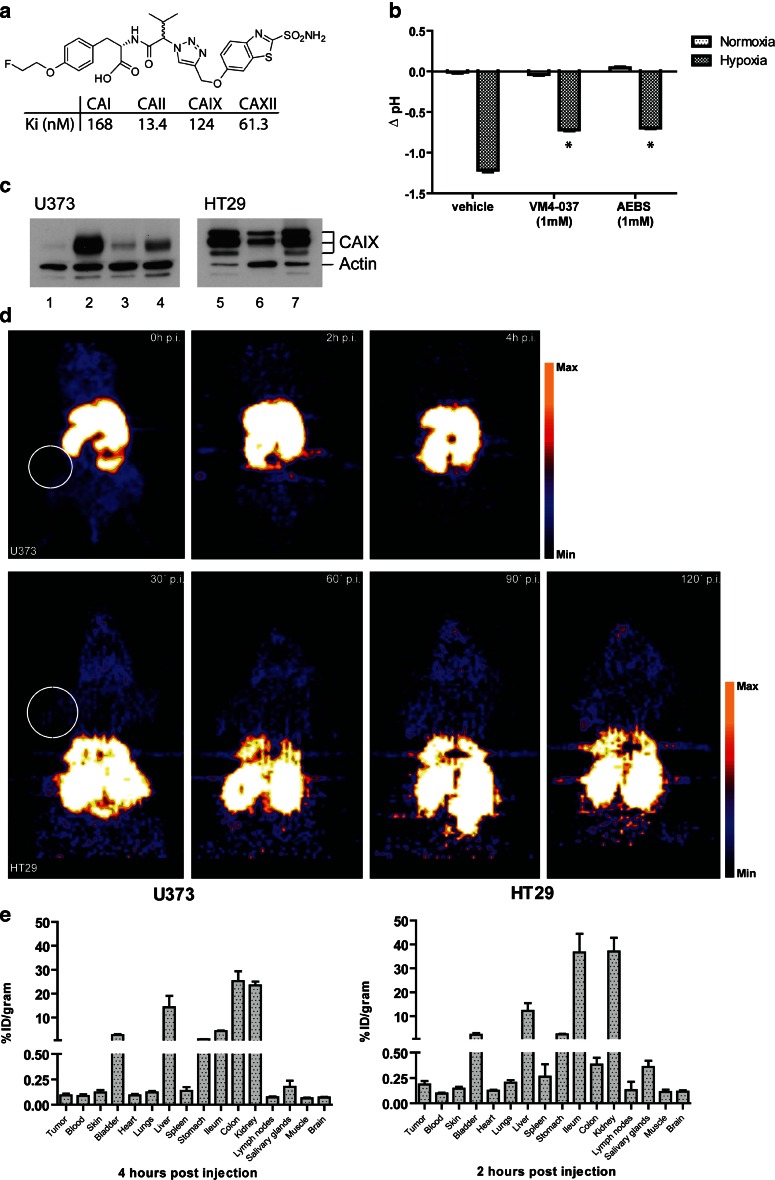
[^18^F]VM4-037 in CAIX expression tumors. **a** Structure formula of VM4-037 with Ki values for CAI, CAII, CAIX, and CAXII. **b** *In vitro* delta pH assay on HT29 cells exposed for 24 h to 0.2 % hypoxia either with vehicle, VM4-037, or the positive control AEBS (*n* = 4). *Bars* indicate mean plus SEM. **c** Western blot analysis of U373 (*number 1–4*) or HT29 (*number 5–7*) protein samples from tumors excised from mice. The bands of CAIX and actin are indicated. **d** microPET images of mice bearing U373 (*upper*, corresponding to Fig. 1b mouse 1) or HT29 (*lower*, corresponding to Fig. 1b mouse 7) tumors at multiple time points. High uptake in *yellow*/*white*, low uptake in *blue* as represented by the *color scale bars*, expressed as Bq/cc. *White circle* indicates location of tumor. **e** Biodistribution at 4 and 2 h p.i. from U373 (*n* = 4) or HT29 (*n* = 4 mice, three of which had a tumor) tumor-bearing mice, respectively. *Bars* indicate mean plus SEM.

The inhibitory constant of VM4-037 was determined for different carbonic anhydrases and showed a Ki of 124 nM for CAIX while for CAI this was 168 nM, CAII 13.4 nM, and for CAXII 61.3 nM (Fig. 1a). A delta pH assay was performed to verify the CAIX-inhibiting potential of VM4-037. Upon hypoxic conditions, the extracellular environment acidifies, and this acidification is reduced upon incubation with either VM4-037 or the positive control AEBS (Fig. 1b). To assess CAIX expression in the *in vivo* tumors, protein levels were evaluated by Western blot. Although there was variation, all tumors expressed a certain level of CAIX (Fig. 1c).

PET acquisition was performed to investigate the distribution of [^18^F]VM4-037 throughout the animal’s body and to detect specific uptake in the tumor (Fig. 1d). At the first time point, the shape of the mouse is clearly visible, and high uptake is seen in the abdominal region. Over time, the overall signal intensity decreased except for the abdominal region. No signal was detected from the tumor region in any of the scans. In the HT29 model, the shape of the animal was also clearly visible, and high signals were detected in the abdominal region. However, no accumulation was observed in the tumor (Fig. 1d).

A biodistribution study performed on the U373 tumor-bearing animals at 4 h p.i. showed that the organs with more than 1 % ID/g [^18^F]VM4-037 uptake were the colon, kidney, and liver, followed by the ileum, bladder, and stomach (Fig. 1e). Disintegrations detected from the tumor were not higher compared to background tissues like the muscle, blood, and skin. Assessing the biodistribution of [^18^F]VM4-037 at 2 h p.i. in the HT29 tumor-bearing animals showed an uptake of above 30 % ID/g in the kidneys, ileum, and tissues and over 1 % ID/g in the liver, bladder, and stomach (Fig. 1e). The percent injected dose per gram of the tumor was in the same range as in the low-uptake organs.

Metabolite analysis of [^18^F]VM4-037 revealed 87.8 ± 1.5 and 43.1 ± 3.0 % of the tracer to be un-metabolized at 15 and 45 min p.i, respectively. Polar metabolites accounted for 11.1 ± 2.0 %. Two apolar metabolites were found in the 45-min samples, accounting for 27.2 ± 1.9 and 17.1 ± 3.6 %.

## Discussion

This study investigated the distribution of [^18^F]VM4-037 in tumor-bearing animals with the aim to determine the specific accumulation in CAIX-expressing tumors. We assessed the uptake of this tracer in two different CAIX-expressing tumor models using microPET and biodistribution.

Doss et al. assessed the distribution of [^18^F]VM4-037 in healthy volunteers [12], demonstrating the safety of this CAIX PET imaging tracer. Consistent with their results, both our microPET imaging and biodistribution data show a high uptake of the tracer in the kidneys and liver at both 2 and 4 h p.i. Doss et al. also found that radiation dosimetry showed high activity in the bladder at 4.8 h p.i. based on voiding models [12]. In mice, this high-uptake organ was detected at 2 h p.i. together with the colon, ileum, and stomach. The difference in the pharmacokinetics of the tracer in humans compared to mice could be explained by a more rapid metabolism and clearance in mice. This may indicate that at later time points, [^18^F]VM4-037 might non-specifically accumulate in other human organs of the abdominal region besides the kidney and liver.

The main goal of our study was to assess the specific uptake of [^18^F]VM4-037 in CAIX-expressing tumors. Although the U373 tumors expressed CAIX, as validated by Western blot, microPET imaging could not distinguish tumor tissue from background tissue. The observed spread in CAIX expression in Fig. 1b is part of the natural variation between the different tumors. A second tumor model with an even higher and more stable CAIX expression was used and was located on the shoulder of the animal preventing the abdominal scatter from interfering with the signal from the tumor. Furthermore, imaging was acquired more frequently in the first 2 h p.i. to ensure coverage of all relevant time points. However, at none of the time points was the tumor distinguishable from the background, suggesting that the tracer does not specifically accumulate in the CAIX-expressing tumors. Biodistribution results were used to clearly distinguish the uptake per organ, especially in the high-uptake regions and the tumor. This confirmed that the uptake of [^18^F]VM4-037 in the tumor was comparable with the non-specific, overall distribution throughout the body of the animals.

Recently, the uptake of [^18^F]VM4-037 was evaluated in kidney cancer patients. Modest uptake signal was detected in the tumors, but no correlation was found with staining for CAIX and CAXII. The authors suggest that larger studies are needed, since in this study only 11 patients underwent imaging with [^18^F]VM4-037 [16]. Although CAIX is a potential marker for clear cell renal cell carcinoma [17], it is remarkable that uptake from the kidney tumors could be distinguished since the current study, as well as the study conducted by Doss et al., showed high non-tumor-related uptake of [^18^F]VM4-037 in this organ.

A new CAIX targeting tracer [^18^F]4d has recently shown to be clearly visible in HT-29 tumors [18]. Furthermore, another CAIX targeting tracer (HE)_3_-ZCAIX:1, based on Affibody molecules, has recently been developed and tested in CAIX-expressing SK-RC-52 renal carcinoma xenografts. The advantage of this new class of molecules is their small size resulting in favorable characteristics regarding extravasation, tissue penetration, and clearance of unbound tracer. These studies indicate that there is a general interest in finding a specific marker to detect CAIX non-invasively [19].

The question remains why no specific tracer accumulation is observed in the tumor. There could be multiple reasons for the discrepancy between the promising *in vitro* results of VM4-037 and the lack of a signal from the tumor *in vivo*. The radiopharmaceutical was >99 % pure at the moment of injection, which means the radiopharmaceutical could not be a reason for the unexpected low tumor uptake. *In vitro*, the CAIX-expressing cells are easy accessible; *in vivo*, however, the bioavailability is influenced by the whole organism. Metabolite analysis showed that 43 % of the tracer was still available in the blood plasma 45 min p.i., which should be sufficient for distribution throughout the body and detection of CAIX at least on the early time points investigated in this study. This indicates that metabolic degradation could not be the cause for the low CAIX specific accumulation. However, characteristics of the tumor microenvironment that are absent *in vitro* could influence the uptake of the tracer in the *in vivo* situation.

## Conclusion

Although [^18^F]VM4-037 demonstrates some activity against CAIX in the *in vitro* delta pH assay and *in vivo* metabolite analysis are favorable, both our *in vivo* microPET and biodistribution results show that the tracer does not specifically accumulate in CAIX-expressing tumors.
